# Provide proactive reproducible analysis transparency with every publication

**DOI:** 10.1098/rsos.241936

**Published:** 2025-03-05

**Authors:** Paul Meijer, Nicole Howard, Jessica Liang, Autumn Kelsey, Sathya Subramanian, Ed Johnson, Paul Mariz, James Harvey, Madeline Ambrose, Vitalii Tereshchenko, Aldan Beaubien, Neelima Inala, Yousef Aggoune, Stark Pister, Anne Vetto, Melissa Kinsey, Tom Bumol, Ananda Goldrath, Xiaojun Li, Troy Torgerson, Peter Skene, Lauren Okada, Christian La France, Zach Thomson, Lucas Graybuck

**Affiliations:** ^1^ Allen Institute for Immunology, 615 Westlake Avenue N, Seattle, WA 98109, USA

**Keywords:** reproducibility crisis, open science, equity in science, data analysis, immunology, life sciences

## Abstract

The high incidence of irreproducible research has led to urgent appeals for transparency and equitable practices in open science. For the scientific disciplines that rely on computationally intensive analyses of large datasets, a granular understanding of the analysis methodology is an essential component of reproducibility. This article discusses the guiding principles of a computational reproducibility framework that enables a scientist to proactively generate a complete reproducible trace as analysis unfolds, and share data, methods and executable tools as part of a scientific publication, allowing other researchers to verify results and easily re-execute the steps of the scientific investigation.

## 1. Introduction

Reproducibility became a focus of debate in the scientific community more than a decade ago, when Amgen researchers revealed that of 53 landmark preclinical cancer studies analysed, the results of only 6 (11%) could be reproduced [1]. In the field of psychology, a similar effort showed that results could not be replicated in one-third to one-half of the 100 studies analysed [2]. A survey of 1500 scientists in chemistry, physics, engineering, earth and environmental sciences, biology and medicine found that, on average across these disciplines, more than 70% failed to reproduce their own or other scientists' findings [3]. These and similar studies show that the inability to reproduce or replicate published results is common and not limited to a particular field [4].

Multiple factors are thought to contribute to this lack of reproducibility or replicability, including poor experimental design, lack of (or poor adherence to) standard operating procedures in the experimental laboratory, poor data analysis, improper interpretation of statistics, unavailability of the original data, selective reporting, the omission of key details about an experiment in published work, shortcomings in the peer review process, a tendency to undervalue studies that fail to reproduce or replicate, the pressure to publish, creative license and outright fraud, as well as other societal and career pressures leading to misconduct [1,3–9]. In response to these findings, the scientific community has widely endorsed open science practices, such as greater transparency in design and analysis and increased sharing of data for examination. Full transparency in experimental design facilitates study replication attempts, where the aim is to generate consistent findings from new data using the original study materials and research methods. Sharing data and methods of analysis is, by definition, necessary for study reproducibility attempts, where the aim is to yield the same results produced by the original research by reanalysing the original data using the same methods. However, even with improved transparency, reproducibility remains difficult in many cases due to incomplete datasets and missing steps in the analysis [10,11].

The reproducibility crisis in science has spurred the development of open science practices. Initiatives like the Center for Open Science, founded in 2013, promote transparency and support the Open Science Framework (OSF) for managing, collaborating on and sharing research (https://osf.io/). The FAIR data principles (Findable, Accessible, Interoperable and Reusable) have gained widespread acceptance [12], influencing data management plans across disciplines, such as neuroimaging [13], GO-FAIR Implementation Networks [14] and shaping National Institutes of Health (NIH) guidelines for data management and sharing [15].

However, the exponential growth of data generation in recent years, particularly in the life and health sciences, presents new challenges. The rise of technologies like single-cell RNA sequencing, next-generation sequencing (NGS) and spatial omics, along with the increasing emphasis on multi-omics integration, has multiplied the complexity of data analysis. These advancements necessitate a re-evaluation and refinement of existing open science practices to effectively address the evolving needs of modern research.

This study focuses on reproducibility as it pertains to data analysis, reporting and dissemination of results in life and health sciences research. We argue that the surge in data generation demands real-time, automated data management to minimize human error and prevent having to reconstruct the analysis steps after completion of the analysis. Furthermore, sophisticated multi-step analysis requires that each step of the analysis is captured in great detail akin to the meticulous recording of research data itself. Open sharing of research findings should include not only the data itself but also the complete provenance of the analysis tools used to generate these findings.

We have developed an open science computing system that allows researchers to track their analysis workflows in real-time. This system automatically creates a snapshot at each step in the analysis—every tool, package, script, data input, intermediate result and output is captured in a branching graph that can be attached to the final paper as a single URL. This digital trace provides the scientific community with both the data, the analysis and the computational environment to allow independent confirmation of the original work. By providing the requisite computing infrastructure to the open science community, this framework eliminates the need for external researchers to establish their own environments, thereby lowering the barrier to entry and ensuring equitable access to research findings.

In developing this open science computing system, we have been guided by the following six principles:

— Ensure proactive transparency
— Track data and transformations
— Streamline administrative overhead through automation
— Promote transparency
— Use executable tooling
— Support open science with equitable access.

Below, we will describe these guiding principles in detail and discuss their implications. Next, we illustrate their application through several case studies using our open science computing system called the Human Immune System Explorer (HISE), an immunology research platform that was founded by the Allen Institute for Immunology in 2019 [16]. Although HISE is specifically designed for immunology and omics research, we contend that analogous platforms can be developed for other scientific disciplines by following these principles while addressing the unique requirements of these fields.

## 2. Ensure proactive transparency

While scientific journals and granting agencies like the NIH strongly advocate for transparency in data and methods, they are less outspoken about the processes that enable such openness [17,18]. A key question regarding achieving research transparency is when these efforts should begin. One approach, often seen as the default, is to address transparency only after research completion and manuscript preparation. This is often because data dissemination might be a requirement for publication. Let us call this the retroactive method. In contrast, a proactive approach advocates for continuous tracking of data and methods throughout the research process. Our computational framework champions the proactive approach.

In a retroactive approach, a scientific team retraces its steps to reconstruct its analysis path. If this backtracking process occurs around the time of manuscript submission, there can be a substantial delay between the actual analysis and its retroactive recording. This delay increases the likelihood of missing key details.

In a proactive approach, the scientific team actively tracks analysis methodology as the study unfolds. This real-time approach creates a more reliable trace of the analysis process. Furthermore, automating trace construction within the framework further enhances reliability. We will discuss the importance of reducing administrative overhead through automation later. Ultimately, by publication time, the framework holds a complete trace that can be readily shared with external scientists for review.

The ongoing push for open science practices is a positive step towards research transparency [19]. However, simply sharing more data and tools does not guarantee completeness. Current practices may result in partially reconstructed analysis traces, making it difficult for other researchers to find, understand and ultimately reproduce the original findings [20,21]. This lack of complete information can limit the ability to verify the research and potentially hinders scientific progress [10,11,22]. While discrepancies in analysis might not always materially impact the conclusions [10,23], championing a proactive approach to obtain a complete trace of the analysis can significantly enhance the trustworthiness and reproducibility of research.

The proactive method focuses not only on achieving openness and reproducibility of published material for external scientists but also on extending this transparency to peers in the laboratory as the research is undertaken. This shift allows for earlier, continuous verification of results, greatly enhancing the likelihood that the eventual published research will include a complete, usable set of data and methods [17,24,25].

## 3. Tracking data and transformations

To ensure a complete audit trail, both the data and the data manipulations must be tracked. This includes capturing raw data, quality control (QC) steps, primary analyses (e.g. per-sample or per-subject), filtering decisions, secondary analyses (e.g. cross-sample or cross-subject), visualizations and any other relevant transformations. By capturing this detailed record, researchers gain complete insight into the exact analytic procedures employed which is imperative for transparency [10], and allows researchers to evaluate the chosen analysis strategy against valid alternative approaches [26–28].

**Figure 1.**
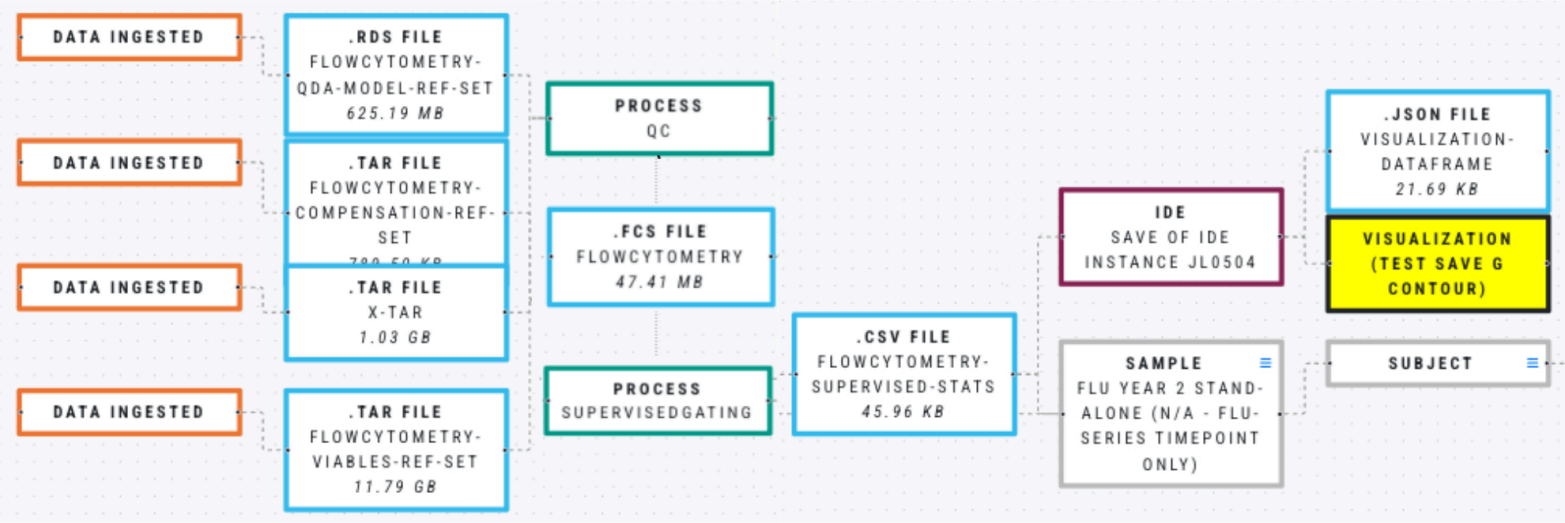
The full provenance of an analysis.

An example of tracking data and transformation is shown in figure 1. This example is from an analysis approach in flow cytometry, a technique used to analyse the physical and chemical properties of a cell. It shows that multiple files were ingested, subjected to a QC process, and then combined into a single flow cytometry standard file. Next, a gating analysis mechanism, which is used to identify and count distinct cell populations, generated a file containing statistical findings. This file was used by an analyst in a coding environment to produce a visualization.

Data immutability—ensuring that the original data and all intermediate results remain unaltered—is paramount for tracking multi-step analyses. Simply storing the raw input and final output is insufficient. Meticulous tracking of the data generated at every step within the analysis pipeline is essential to accurately track provenance. This rigorous data management policy is crucial in maintaining the integrity of the research trace.

To ensure the reproducibility of data transformations, it is essential to meticulously record the full execution details of each step [29,30]. This comprehensive record should encompass:

— *Code and configuration*: The exact code used for the transformation, along with any configuration files or settings that define its behaviour.
— *Dependent libraries*: A list of all software libraries the code relies on, including their specific versions.
— *Runtime environment*: A description of the computational environment where the code was executed, including the operating system, hardware specifications and any relevant software versions.
— *Data dependencies*: Information about the reference datasets or trained models used in the transformation step.
— *Additional components*: Any other elements crucial for reproducing the computational environment, such as custom scripts or tools.

The platform we have developed creates a comprehensive record of a transformation by implementing orthogonal software best practices that capture the runtime details of running an analysis. These best practices include version control of source code, use of systems like Docker containers to modularize runtime components and ensure consistent runtime environments, and reliance on library management systems like Conda to accurately record the specific versions of the libraries and dependencies used.

**Figure 2.**
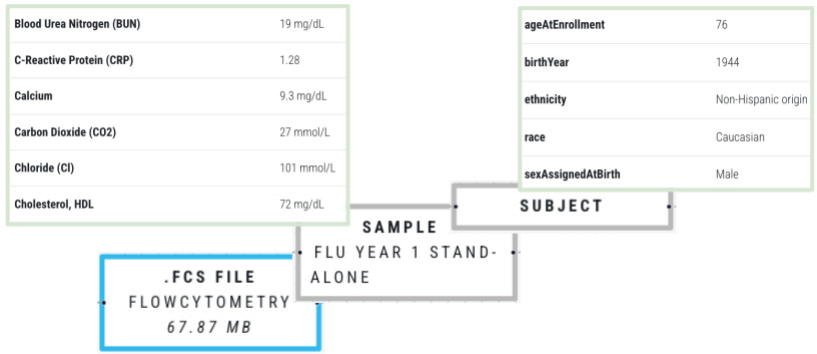
An example of subject metadata associated with a dataset.

Capturing detailed metadata associated with the subject or sample, the data itself and the transformation process further enhances the interpretability of the trace. Subject and sample metadata describing the (non)human subjects and specimens under study must be included in the full dataset as this will affect the interpretation of the results [4]. Figure 2 shows an example of metadata associated with the data and includes subject demographics and specifics of sample collection.

Metadata on the data itself may include creation date, storage information (e.g. location and file type), data format (e.g. CSV or H5) and data modality (e.g. microscopy image, single-cell RNA sequences or reaction time data). This comprehensive information helps researchers understand the origin, format and nature of the data, ultimately facilitating accurate analysis and interpretation of the results.

**Figure 3.**
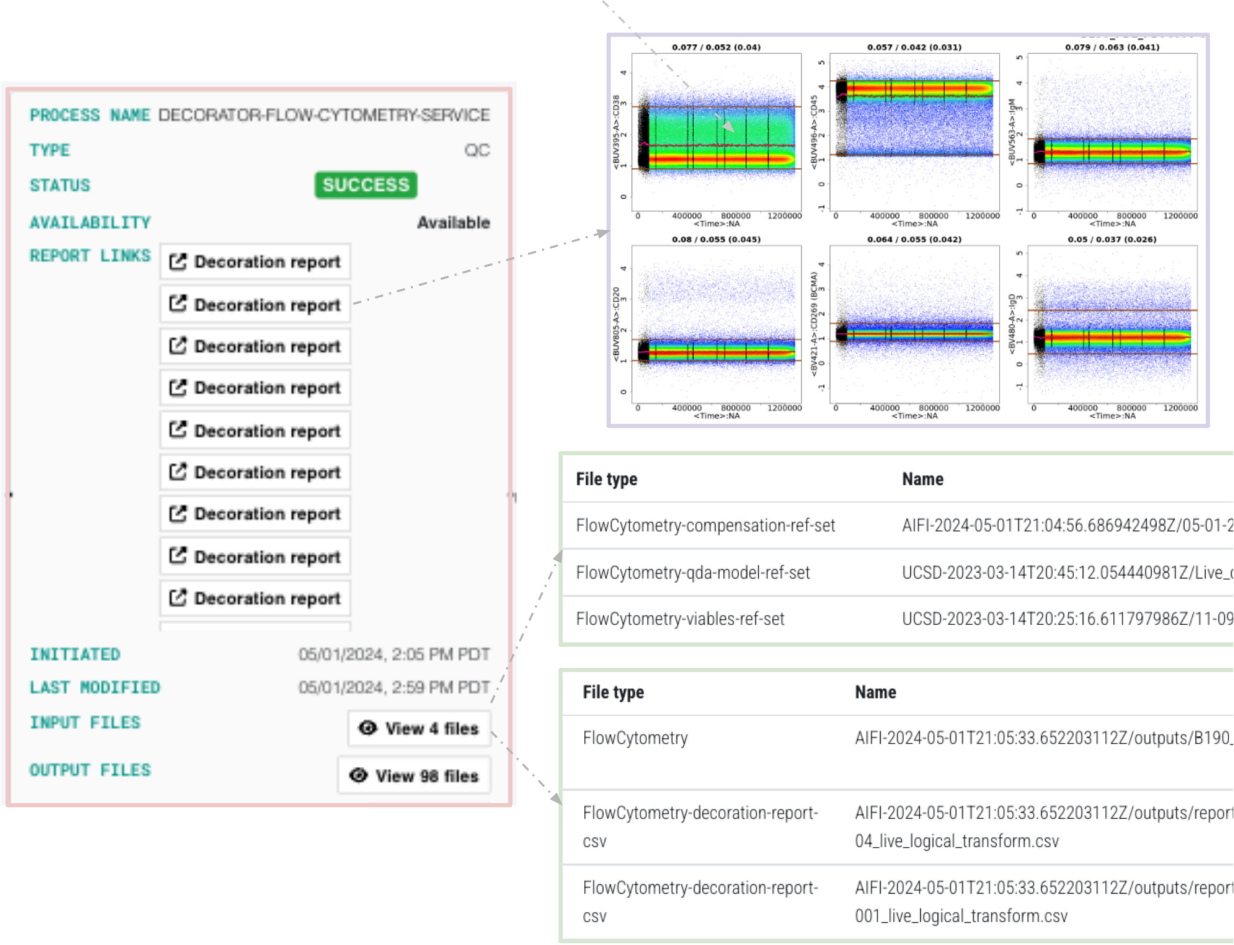
An example of structural metadata for a transformation step.

Finally, structural metadata on the transformation process, including input data, transformation type and output data, creates a connected trace by providing the critical links between data and its transformations. Figure 3 shows an example of a transformation process, with insets highlighting specific steps.

**Figure 4.**
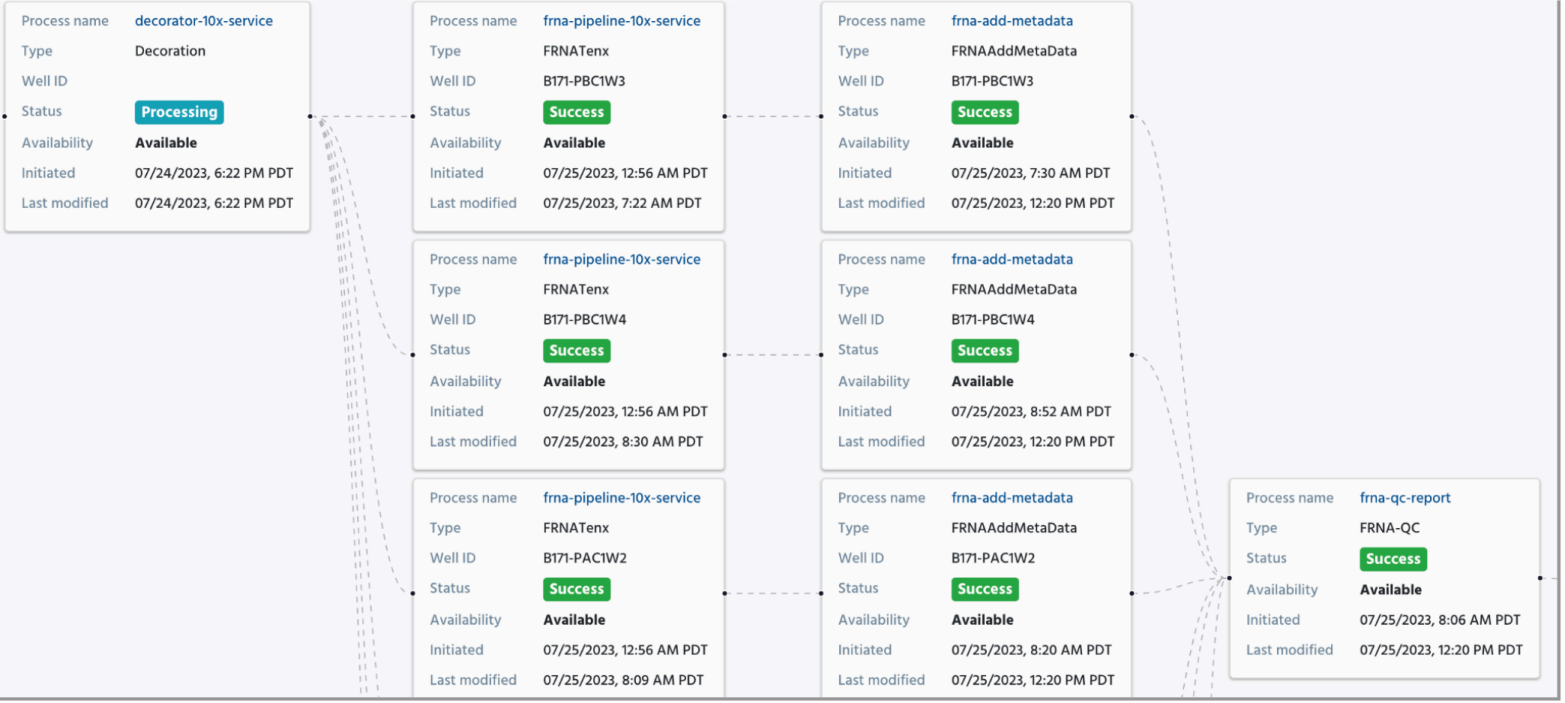
An example of a partial trace of a set of related processes.

In complex analysis pipelines, where the output from one step becomes the input for the next, an intricate network of interconnected processes emerges. Figure 4, derived from a standardized pipeline within HISE detailed further in the ‘Case Studies’ section, exemplifies this approach. HISE incorporates a collection of standardized pipelines employed across numerous research studies to analyse unprocessed batch or sample data. Figure 4 specifically illustrates data analysis utilizing a standardized single-cell RNA sequencing technique.

We contend that robust metadata management must be an integral component of any sound research management policy. In our experience, this critical aspect of research is frequently overlooked or inadequately addressed. At the outset of each study, researchers should establish an agreed-upon data dictionary to outline the essential metadata elements in granular detail. While we acknowledge the value of discipline-specific standards, their existence is not a pre-requisite for effective metadata management. In the context of immunology, a field in which standardized metadata frameworks are currently lacking, our HISE system is designed to enforce consistent metadata capture and utilization within individual research endeavours, without imposing a universal standard across all immunological research studies.

## 4. Streamline administrative overhead through automation

Adhering to open science principles by providing insights into data and methods can add to the administrative workload of the researcher. Other open science initiatives have shown that this bureaucratic burden may obstruct adoption and follow-through [4]. In the proactive approach, lowering administrative overhead is essential for adoption, as all analyses are tracked whether or not they lead to publication. Automating as much of the tracing process as possible is critical to lowering overhead and encouraging researchers to adopt the approach. In the computational framework described here, researchers' actions, such as data access and running computations, are mediated through an abstraction layer. This layer enables machine processing for tracking their actions. Analysts' manual input is limited to key moments in the process, such as selecting specific data subsets for analysis or defining parameters for generating new results.

Proactive, largely automated tracing not only reduces overhead during analysis but also reduces the burden of support after publication. Research on the analysis reproducibility of published studies that adhere to open science practices shows that in one-quarter to nearly one-half of the studies, reproducibility could be achieved only after authors provided more detailed information about their tools and workflow [10,23]. Eliminating or reducing the need to contact authors to request more information will reduce interruptions and should significantly improve the completion rate of reproducibility attempts.

## 5. Promote transparency

Traces should be part of an open science release along with a badge verifying that the work is fully transparent. For instance, a trace that provides the entire flow—from raw data acquisition to automated pipeline analysis to the creation of derived results through secondary analysis, including the creation of visualizations to understand those insights—is more transparent than a trace that offers only visualization insights on derived results. For instance, in HISE, a *certificate of reproducibility* signifies that published research adheres to transparent workflows, ensuring the analysis reproducibility of its findings. This certificate of reproducibility is made available on the HISE open science portal along with the biological insights to allow the scientific community to verify the results. The ability to earn badges and display them to external scientists has been shown to be effective in promoting open science practices [11].

To further promote and reward transparency, the data and the underlying infrastructure provided with the results should be recognized as its own scholarly output [31]. To that end, in HISE each release with a certificate of reproducibility is assigned a digital object identifier (DOI) to easily locate it on the open science portal and to enable its direct citation and recognition as a separate body of academic work.

Releasing a trace to the science community such as the certificate of reproducibility in HISE signifies that data analysis was conducted using a specific set of analytical tools. Importantly, this does not necessitate the exclusive use of uniquely developed tools. To uphold the principles of open science transparency, any novel analysis methods or libraries created during a research study, that have potential for broader applicability, should be disseminated through open-source channels. Furthermore, when warranted, a separate scientific publication should be considered to formally present these novel analysis methods and highlight their scientific significance.

## 6. Use executable tooling

To re-execute data analysis for verification, it is essential that the complete computational infrastructure be made available [29,30,32]. In our reproducibility framework, a certificate of reproducibility containing the full trace, a badge and a DOI can be visually inspected by any scientist, meaning that the steps in the analysis can be reviewed. Additionally, an outside scientist can re-execute (portions of) the trace. For instance, they may opt to re-run a secondary analysis that was originally run in a Jupyter Notebook. To avoid variation introduced by the setup of a scientist’s local machine, the scientist is provided the full virtual infrastructure containing the original coding environment of that notebook, including the original (virtual) machine, the (scientific) libraries used, the code and the input data for that analysis. In the same way, if the user wants to re-run an analysis pipeline, this can be done in the provided infrastructure.

Several software mechanisms are available to achieve executable tooling, and new mechanisms will undoubtedly emerge in the future. In an HISE open science release, such as those detailed in the subsequent ‘Case Studies’ section, the certificate of reproducibility can be re-executed either comprehensively or by selectively replaying steps of analysis. To facilitate this exploration, open science users are invited to participate in a process that generates a cloud-based, sandboxed environment within HISE that reproduces the complete runtime environment necessary to re-execute the certificate. The underlying software mechanisms employed—which currently include Docker containers, Argo orchestration and Kubernetes container management—are abstracted away from the user, thereby lowering the bar to entry for reproducing the findings.

## 7. Support open science with equitable access

Adherence to open science principles is a central tenet of our reproducibility framework. The chief self-correcting mechanism of science is the scientific community’s engagement in debate and evaluation of findings [33]. To facilitate this debate and easily detect and eliminate implicit assumptions, published research must be accessible and interpretable by a diverse scientific community [33,34]. Scientific analysis increasingly relies on considerable resources, such as the infrastructure needed to support computational, storage and other resources and the expertise needed to operate this infrastructure. Given that there are structural inequities between scientists and institutions with regard to these resources [35], providing open access to scientific analysis cannot be limited to making the data available but must also include access to the tools and resources needed to verify results [36].

Our reproducibility framework is designed to ensure equitable access for all outside scientists. By enabling direct reproduction and evaluation of results within HISE using the provided tools, we significantly lower the financial and technological barriers to entry. This eliminates the need for researchers to establish and maintain their own complex infrastructure, which would demand substantial resources, expertise and ongoing maintenance efforts.

## 8. Case studies

Next, we examine several studies run and analysed using HISE [16]. The HISE was founded in 2019 by the Allen Institute for Immunology based on the guiding principles described above. It is designed to support the Institute’s research into the human immune system in health and disease. The Allen Institute for Immunology works closely with research institutes, hospitals and pharmaceutical companies to understand immune responses. This research focuses heavily on multi-year longitudinal studies with healthy subjects and patient populations, using a multi-omics deep immune profiling approach to address our research questions.

As a multi-tenant cloud-based SaaS application, HISE has been instrumental in numerous research initiatives. The platform manages petabytes of research data and facilitates thousands of hours of high-performance scientific computing each week. It features both a registered-user component for collaborative research among cross-disciplinary and cross-organizational scientists, as well as an open science community space for sharing data, tools, runtime environments and biological insights with the broader scientific community.

The case studies described here are only two illustrations of the research that HISE supports. For other research that has been published to its open science portal, please visit https://explore.allenimmunology.org. Both case studies presented here utilize data previously described in [37].

The study ‘Comparison of Leukapheresis PBMC and Ficoll PBMC scRNA-seq’ (https://doi.org/10.57785/96bw-7571) showcases the HISE provenance tracing functionality, starting with automated pipeline processing of data from two single-cell RNA sequencing (scRNA-seq) samples generated using different cell isolation methods for peripheral blood mononuclear cells. Processing produced labelled scRNA-seq files in HDF5 (.h5) format suitable for data analysis. The authors used HISE to provision a cloud-based Jupyter Notebook analysis environment to perform a comparative analysis of these data, generating a dataset that summarizes the results. Along with this dataset, HISE captured and stored the exact analysis environment and Jupyter Notebook code used for this analysis for later inspection, reproduction or modification. The certificate of reproducibility demonstrates the use of a standardized pipeline as a primary analysis step, followed by the use of a Jupyter Notebook to perform secondary, cross-sample analysis.

In the study ‘Comparison of Pediatric and Older Adult T cells from TEA-seq’ (https://doi.org/10.57785/sv3w-w848), the single-cell tri-modal genomics method, TEA-seq (for Transcriptomics, chromatin Accessibility and surface Epitopes), was used to characterize the T cells from the blood of healthy paediatric and young adult samples (*n* = 4 for each group). The authors employed the reproducibility framework to perform and capture a branching, multi-step data analysis workflow, generating a dataset and an interactive visualization to showcase the results. A certificate of reproducibility offers complete transparency into this analytical process. As the provenance trace highlights, the authors adopted a modular approach to analysis. This involved breaking down the analysis into discrete steps, each executed within a separate Jupyter Notebook, and each with its corresponding analysis environment captured in HISE. By persisting all intermediate results, the authors ensured the ability to revisit and review each step independently. This approach not only facilitates exploration of alternative algorithmic approaches during analysis but also makes the work more transparent and verifiable by the open science community. Since each step is self-contained, it can be easily assessed, modified and rerun.

By showcasing HISE and its successful application in two distinct research scenarios, we demonstrate the effectiveness of our guiding principles in supporting complex and demanding scientific investigations.

## 9. Discussion

The above case studies demonstrate that utilizing an open science computational framework not only enhances comprehension of research but also deepens insights. This framework, along with its guiding principles, can serve as a valuable blueprint for numerous organizations seeking to achieve analysis reproducibility for many organizations. Full support for open science practices, however, requires a multi-pronged approach that goes beyond technological solutions.

### 9.1. Failures versus flaws

One issue debated within the open science community concerns the interpretation of failed reproduction attempts. Are these failures solely indicative of errors in the original research, or do they present opportunities to uncover hidden variables and refine our understanding of the phenomenon under investigation?

Reproducibility is a multi-faceted concept, and an inability to reproduce a particular study may hint at scientifically relevant—albeit perhaps nuanced—differences between the original and the reproduced analysis. If seemingly minor variations in data pruning or analysis method lead to a failure to reproduce the original study’s findings, this can help uncover hidden confounding variables and stimulate further scientific debate. This is evident when slight alterations in data cleaning drastically alter the significance of findings, or when commonly used alternative analysis methods, such as the diverse approaches to assessing statistical significance with Likert scale data, produce substantially different outcomes. In these cases, the scientific debate unfolds as studies build on each other—a form of reproducibility essential to scientific progress [33,38]. This phenomenon has been described as a scientific *failure* [39].

Alternatively, the inability to reproduce study results may be caused by errors or mistakes unwittingly introduced either during execution of the original research or during the reproducibility attempt. This phenomenon, which has been described as a research *flaw*, detracts from the larger scientific debate [39].

The open science frameworks proposed in this article make it easier to detect scientific flaws both before publication and in published work. They also help reveal scientific failures by delineating the analytical approach and facilitating a debate on alternative analytic approaches that may produce different outcomes.

### 9.2. Rewarding open science as an organizational practice

A key incentive for scientists' active adherence to open science practices is that organizations actively reward and recognize open and responsible research. This includes investing in the resources, infrastructure and training to effectively share data and methods within and outside the research laboratory [17,24,40]. Scientists are more motivated to adopt a computational reproducibility framework based on the guiding principles presented in the current study when research organizations encourage open science and team science practices. Unfortunately, such support appears to be insufficient in many research institutions and academic organizations, and more must be done to encourage it [36].

### 9.3. How free is FAIR and equitable access?

The cost of non-reproducible research is considerable. An estimated $25 billion is spent annually on non-reproducible studies in preclinical research alone [41]. In the Freedman *et al* (2015) study, the authors argue that although implementing measures to improve reproducibility would raise the cost of individual studies, reducing the percentage of non-reproducible studies will yield significant cost savings. An approach, as chosen in the current study where a trace is created and made available as analysis unfolds, enables scientific flaws to be caught early, before publication and limits the release of non-reproducible research.

Conversely, implementing an open science approach requires substantial funding and resource investments. To effectively support an organization’s long-term commitment to transparent research practices, establishing a cost recovery mechanism is essential [40]. Securing long-term financial stability through proper funding and resource allocation is essential for both ensuring internal adherence to open science best practices and earning the recognition and trust of external researchers in the data, tools, and technology provided.

HISE offers a comprehensive package: access to the underlying framework itself, secure data storage for research materials, and the necessary computational resources for re-running analyses. Notably, our framework prioritizes equitable access for external researchers. When reproducing analyses, they incur only the cloud infrastructure costs directly associated with their specific storage and computations. Although this is not entirely free, open science visitors to HISE are not required to bear the costs associated with the HISE platform itself. Instead, they are responsible only for the expenses directly incurred during the re-execution of the certificate of reproducibility. This approach represents a significant reduction compared with the substantial financial and resource investment that would be necessary for researchers to independently establish and maintain the requisite computing infrastructure.

The science community should consider two primary funding models to sustain open science practices long-term: a provider-pays model and a user-pays model. In the provider-pays model, the provider of the open reproducibility infrastructure would incorporate the associated costs into their grant applications. While this approach would necessitate a paradigm shift in how funders view and allocate research resources for open science, it could offer a pathway for sustainable support. Alternatively, a user-pays model could be implemented, in which researchers who use the open reproducibility infrastructure would be responsible for a nominal fee to cover operational expenses. This fee could be offset by inclusion into their grant applications.

### 9.4. Analysis reproducibility and study reproducibility

Analysis reproducibility is one of the components contributing to full reproducibility. In life sciences, data generation and collection in the wet laboratory are usually a major focus [3,4]. For example, for our studies in human immunology, major efforts were undertaken to develop standard operating procedures for blood and tissue sample procurement and processing at different geographic sites, including setting tight standards on processing delays [42,43]. Although a full discussion of best practices in the wet laboratory is outside the scope of the current study, it should be noted that standardization of data collection in the wet laboratory enables downstream standardization and greatly facilitates data ingestion and uniform analysis.

Another important facet of open science is promoting collaborative research. A team science approach, emphasizing collaboration from the study’s conception and continuing throughout all phases, enhances reproducibility [44,45]. Team science can be amplified by providing a common platform that fosters transparency by allowing all team members to collaborate on study design and analysis, getting direct access to results and monitoring progress. Several specialized team science platforms exist, such as the Open Science Framework [46] and the HISE [16], which we discuss here in the context of its analysis reproducibility design principles.

### 9.5. Interoperability limits

Effective integration of third-party analysis tools within reproducibility frameworks like HISE requires adherence to specific principles. Crucially, these tools must minimally support the tracking of data and transformations. This implies that the input data used for analysis must be clearly identifiable and that the mechanism employed to generate the output data should be both trackable and replayable. If the analysis tool’s transformation mechanism lacks transparency, for example due to manual inputs that are not captured within the system, provenance could be significantly compromised.

Furthermore, seamless interoperability demands that the analysis tool be compatible with the deployment mechanisms utilized by HISE. For instance, research software confined to desktop environments lacks containerization capabilities and cannot be effectively integrated into a cloud infrastructure.

By advocating for this updated approach to reproducibility, we aim to encourage the development of software tools that prioritize these key principles, thereby enhancing the overall reproducibility and interoperability within the scientific research ecosystem.

### 9.6. Reproducibility and public trust in science

Supporting full adoption of the open science principles proposed here is essential to the continued success and recognition of science in all its manifestations, including fundamental and applied science, both publicly and privately funded. Fundamental science, the basic research that many believe is an essential precursor to breakthrough innovation, is sensitive to public funding cuts, as the usefulness of non-applied research is often questioned by the general public [47]. Improving reproducibility would help boost trust in basic research and bolster the case for continued strong public funding. Similarly, withholding the findings of privately funded applied science has hampered and delayed scientific debate on issues ranging from the effects of tobacco smoke to global warming, undermining trust in industry-funded research [48,49]. In both cases, adherence to open science principles and encouragement of open debate would go a long way toward restoring and strengthening public trust in science.

## 10. Conclusion

Open science principles are fundamental to enhancing research reproducibility and fostering trust in scientific findings. Recognizing the significant increase in data generation and complexity of analysis in health and life sciences research, we believe that fostering proactive data tracing is crucial for promoting analysis reproducibility. The guiding principles of the reproducibility framework we present here aim to limit scientific flaws, enable easy detection of scientific failure and can spur a discussion of alternative analysis approaches while ensuring equitable access for all members of the academic community.

Universities, research organizations and granting agencies play a pivotal role in fostering and rewarding open science and collaborative team science practices in all phases of research. As Big Data and intense computation become increasingly commonplace in science, equitable access to data, methods and computing infrastructure is essential to ensure the participation of the entire scientific community. By actively supporting these practices, they can significantly contribute to the advancement of reliable and trustworthy scientific knowledge.

## Data Availability

The scientific case studies used to demonstrate Certificates of Reproducibility were generated from data originally described in [37]. Human samples for this study were collected following protocols approved by the IRB of Benaroya Research Institute (Older Adult samples) and the IRB of the Children's Hospital of Philadelphia (Paediatric samples). Raw data are deposited indbGaP for controlled access at dbGaP Study ID phs003400.v1.p1. Processed data are openly available in NCBI GEO at GEO Accession GSE214546. The code used to generate these case studies is available on Github at [50].
